# Unravelling the genomic architecture of bull fertility in Holstein cattle

**DOI:** 10.1186/s12863-016-0454-6

**Published:** 2016-11-14

**Authors:** Yi Han, Francisco Peñagaricano

**Affiliations:** 1 Department of Animal Sciences, University of Florida, 2250 Shealy Drive, Gainesville, FL 32611 USA; 2 University of Florida Genetics Institute, University of Florida, Gainesville, FL 32610 USA

**Keywords:** Bovine sperm, Conception rate, Gene set analysis, Whole-genome scan

## Abstract

**Background:**

Fertility is considered an important economic trait in dairy cattle. Most studies have investigated cow fertility while bull fertility has received much less consideration. The main objective of this study was to perform a comprehensive genomic analysis in order to unravel the genomic architecture underlying sire fertility in Holstein dairy cattle. The analysis included the application of alternative genome-wide association mapping approaches and the subsequent use of diverse gene set enrichment tools.

**Results:**

The association analyses identified at least eight genomic regions strongly associated with bull fertility. Most of these regions harbor genes, such as *KAT8*, *CKB*, *TDRD9* and *IGF1R*, with functions related to sperm biology, including sperm development, motility and sperm-egg interaction. Moreover, the gene set analyses revealed many significant functional terms, including fertilization, sperm motility, calcium channel regulation, and SNARE proteins. Most of these terms are directly implicated in sperm physiology and male fertility.

**Conclusions:**

This study contributes to the identification of genetic variants and biological processes underlying sire fertility. These findings can provide opportunities for improving bull fertility via marker-assisted selection.

**Electronic supplementary material:**

The online version of this article (doi:10.1186/s12863-016-0454-6) contains supplementary material, which is available to authorized users.

## Background

Improving reproductive efficiency of dairy cattle has become one of the major challenges of the dairy industry worldwide. The intense selection for production traits in the last decades has led to a decrease in fertility [1, 2]. Fertilization failure and early embryonic loss have been identified as the two main factors contributing to this decline [3, 4]. For instance, fertilization rate in high-producing dairy cows is about 75 %, and only 65 % of the fertilized eggs are considered viable at 5–6 days post-fertilization [5]. It is no surprise that conception rates are only 35–45 % [5]. Many reasons may account for this decline in reproductive performance, including physiological, nutritional, environmental, and genetic factors. In this sense, several studies have recognized that there is substantial genetic variation underlying reproductive success in dairy cattle [6, 7].

Reproduction is a very complex process that involves numerous consecutive events, including gametogenesis, fertilization, and early embryo development, that should be accomplished in a well-orchestrated manner in order to achieve a successful pregnancy. The relative importance of the parental effects on the reproductive success, i.e., maternal versus paternal contribution to the zygote, is still largely unknown [8]. Most studies in dairy cattle have focused on female fertility, while male fertility has received much less attention. It is worth noting that the service sire has a direct influence not only in the fertilization process but also on the viability of the preimplantation embryo [9, 10]. In fact, previous studies have reported that the service sire represents an important source of variation for conception rate in dairy cattle [11–13].

Both candidate gene [14–16] and whole-genome scan [17–21] approaches have attempted to identify genomic regions and individual genes responsible for the genetic variation in bull fertility. For instance, two highly conserved spermatogenesis genes, *MAP1B* and *PPP1R11*, were significantly associated with male fertility in Holsteins [16]. In addition, genetic markers in BTA2, BTA5, BTA14, and BTAX were associated with testicular development, sperm quality, and hormone levels in young Brahman and Tropical Composite bulls [19, 21]. It should be noted that these association studies detect in general only the most significant markers, and hence, the vast majority of the genetic variants contributing to the trait remains hidden. In this context, gene set or pathway-based analysis offers an alternative strategy based on evaluating modules of functionally related genes, rather than focusing only on the most significant markers [22, 23]. This approach provides unique opportunities to detect the genetic mechanisms underlying complex phenotypes. Indeed, using this pathway-based approach, we have identified some processes, such as small GTPases mediated signal transduction or calcium ion binding, that may explain part of the differences in sire fertility [24].

The main objective of this study was to unravel the genomic architecture underlying sire fertility in dairy cattle. Sire Conception Rate (SCR) was used as a measured of bull fertility. SCR is a new and more accurate phenotypic evaluation of dairy sire fertility calculated using field data. Two complementary genome-wide association approaches plus different gene set analyses were performed in order to identify genomic regions, individual genes, functional gene terms, and biological pathways associated with sire fertility. These findings can contribute to a better understanding of the genetics underlying this complex trait and may point out opportunities for improving bull fertility via selective breeding.

## Methods

### Phenotypic and genotypic data

The Animal Improvement Programs Laboratory of the United States Department of Agriculture (AIPL-USDA) implemented in 2008 a national phenotypic evaluation of bull fertility called Sire Conception Rate (SCR). The model that is being used in the U.S. bull fertility evaluation includes both factors related to the service sire under evaluation (including age of the bull and AI organization) and also factors (nuisance variables) associated with the cow that receives the unit of semen (including herd-year-season, cow age, parity, and milk yield) [25, 26]. The trait SCR is defined as the expected difference in conception rate of a given bull compared to the mean of all other evaluated bulls; in other words, a bull with an SCR value of +5.0 % is expected to achieve a conception rate of 37 % in a herd that normally averages 32 % and uses average SCR bulls. It is worth noting that the U.S. bull fertility evaluation, in contrast to evaluations for other traits such as production, is intended as a phenotypic rather that a genetic evaluation, because the estimates include not only genetic but also some (permanent) environmental effects.

The entire evaluation of U.S. Holstein bull fertility was used in this study. Specifically, a total of 44,449 SCR records were available from a total of 10,884 Holstein bulls. These SCR records were obtained from 23 consecutive evaluations provided to the U.S. dairy industry between August 2008 and April 2016. These 23 different SCR evaluations are available at the Council of Dairy Cattle Breeding (CDCB) website (https://www.cdcb.us/). Additional file 1 shows (A) the distribution of SCR values per evaluation and (B) the distribution of the number of SCR records per bull, i.e., total number of repeated measurements per sire evaluated. The reliabilities of the SCR records, calculated as a function of the number of breedings, were also available for the analyses.

Genotype data for 60,671 single nucleotide polymorphism (SNP) markers were available for 7447 out of the 10,884 Holstein bulls with SCR evaluation. The SNP data were kindly provided by the Cooperative Dairy DNA Repository (CDDR). Those SNP markers that mapped to the sex chromosomes, or were monomorphic, or had minor allele frequency less than 1 % were removed from our dataset. After data editing, a total of 58,029 SNP markers were retained for subsequent genomic analysis.

### Statistical methods for genome-wide association mapping

The association analysis between phenotypes and genotypes using related individuals with repeated measurements can be implemented within the framework of the classical repeatability animal model,

$$ \mathbf{y}=\mathbf{X}\boldsymbol{\upbeta } +\mathbf{Z}\mathbf{u}+\mathbf{W}\mathbf{p}\mathbf{e}+\mathbf{e} $$

where **y** is the vector of phenotypic records (SCR values), **β** is the vector of fixed effects included in the model, **u** is the vector of random animal effects, **pe** is the vector of random permanent environmental and non-additive effects, and **e** is the vector of random residual effects. The matrices **X**, **Z**, and **W** are the incidence matrices relating phenotypic records to fixed, animal, and permanent environmental effects, respectively. In this context, the random effects are assumed to follow a multivariate normal distribution,

$$ \left(\left.\begin{array}{c}\hfill \mathbf{u}\hfill \\ {}\hfill \mathbf{p}\mathbf{e}\hfill \\ {}\hfill \mathbf{e}\hfill \end{array}\right|{\sigma}_u^2,{\sigma}_{pe}^2,{\sigma}_e^2\right)\sim N\left[\mathbf{0},\left(\begin{array}{ccc}\hfill \mathbf{K}{\sigma}_u^2\hfill & \hfill \mathbf{0}\hfill & \hfill \mathbf{0}\hfill \\ {}\hfill \mathbf{0}\hfill & \hfill \mathbf{I}{\sigma}_{pe}^2\hfill & \hfill \mathbf{0}\hfill \\ {}\hfill \mathbf{0}\hfill & \hfill \mathbf{0}\hfill & \hfill \mathbf{R}{\sigma}_e^2\hfill \end{array}\right)\right] $$

where *σ*_*u*_^2^, *σ*_*pe*_^2^, and *σ*_*e*_^2^ are the animal additive genetic, permanent environmental, and residual variances respectively; **K** is a kinship matrix that can be calculated using either pedigree or genotypic information, and **R** is typically an identity matrix (**I**) or a diagonal matrix.

In this particular study, two alternative genome-wide association mapping approaches were performed: (1) single-step genomic best linear unbiased prediction (ssGBLUP) and (2) classical genome-wide association study (cGWAS) using regular single-marker regression analysis but with correction for population structure. The ssGBLUP combines all the available phenotypic, pedigree and genotypic information, and fits all the SNP simultaneously, while cGWAS typically uses only animals that have both phenotypic and genotypic data, and fits the SNP markers one at a time.

#### Genome-wide association mapping using ssGBLUP

The ssGBLUP method is one of a group of statistical methods that were originally developed for genomic prediction and later were extended for performing gene mapping. Indeed, ssGBLUP model is a modification of the classical BLUP model where the pedigree relationship matrix **A** is replaced by **H** which combines pedigree and genotypic information [27]. The combined pedigree-genomic relationship matrix **H** ^− 1^ is calculated as follows,

$$ {\mathbf{H}}^{-1}={\mathbf{A}}^{-1}+\left[\begin{array}{cc}\hfill 0\hfill & \hfill 0\hfill \\ {}\hfill 0\hfill & \hfill {\mathbf{G}}_1^{-1}-{\mathbf{A}}_{22}^{-1}\hfill \end{array}\right] $$

where **G**_1_^− 1^ is the inverse of the genomic relationship matrix and **A**_22_^− 1^ is the inverse of the pedigree-based relationship matrix for genotyped animals. In this case, **G**_1_ has dimensions 7,993 × 7,993 and it was created using the 7447 sires with both SCR and SNP data plus 546 genotyped sires with no SCR records. In addition, the **A** matrix (25,075 × 25,075) was calculated based on a five generation pedigree downloaded from AIPL-USDA website. The random effects were assumed multivariate normal with **u** ∼ *N*(0, **H** *σ*_*u*_^2^), **pe** ∼ *N*(0, **I**_***n***_ *σ*_*pe*_^2^), and **e** ∼ *N*(0, **Q**_*N*_^− 1^ *σ*_*e*_^2^). Note that in this case the original kinship matrix **K** is replaced by **H,** and the residual matrix **R** is the inverse of a diagonal matrix **Q** with its elements representing the reliabilities of the SCR values. The subscripts *n* and *N* indicate the size of the matrices and represent the number of individuals with SCR records (*n* = 10, 884) and the total number of SCR records (*N* = 44, 449), respectively.

Candidate regions associated with sire fertility were identified based on the amount of genetic variance explained by 1.5 Mb window of adjacent SNPs evaluated across the entire bovine genome. Given the genomic estimated breeding values (GEBVs), the SNP effects can be estimated as **ŝ** = **DZ**′[**ZDZ**′]^− 1^ **â**_**g**_, where **ŝ** is the vector of SNP marker effects, **D** is a diagonal matrix of weights of SNPs, and **â**_**g**_ is the vector of GEBVs [28]. The percentage of genetic variance explained by a given 1.5 Mb genomic region was then calculated as,

$$ \frac{Var\left({u}_i\right)}{\sigma_u^2}\times 100=\frac{Var\left({\displaystyle {\sum}_{j=1}^B}{Z}_j{s}_j\right)}{\sigma_u^2}\times 100 $$

where *u*_*i*_ is the genetic value of the *i* ^*th*^ genomic region under consideration, *B* is the total number of adjacent SNPs within the 1.5 Mb region, and *s*_*j*_ is the marker effect of the *j* ^*th*^ SNP within the *i* ^*th*^ region. All the ssGBLUP calculations were performed using the BLUPF90 family of programs from Ignacy Misztal and collaborators, University of Georgia.

#### Genome-wide association mapping using single marker regression (cGWAS)

For the whole genome single marker regression, we extended the repeatability model as,

$$ \mathbf{y}=\mathbf{X}\boldsymbol{\upbeta } +{X}_{SNP}{\beta}_{SNP}+\mathbf{Z}\mathbf{u}+\mathbf{W}\mathbf{p}\mathbf{e}+\mathbf{e} $$

where *X*_*SNP*_ is the design matrix for the SNP under study (coded as 0, 1 or 2) and *β*_*SNP*_ is the regression coefficient or SNP effect (also known as the allele substitution effect). In this particular case, the distribution of the random effects were assumed multivariate normal with **u** ∼ *N*(0, **G**_2_ *σ*_*u*_^2^), **pe** ∼ *N*(0, **I**_***m***_ *σ*_*pe*_^2^), and **e** ∼ *N*(0, **I**_*M*_ *σ*_*e*_^2^). Here the original kinship matrix **K** is replaced by **G**_2_ that is calculated based on the 7447 sires that had both SCR records and genotypic data. The subscripts *m* and *M* indicate the size of the identity matrices and represent the number of individuals with SCR records (n = 7,447) and the total number of SCR records (*N* = 32, 590) used in this particular analysis.

Note that the extended repeatability model can be written as **y** = **Xβ** + *X*_*SNP*_ *β*_*SNP*_ + **ϵ,** where **ϵ** ∼ *N*(0, **V**) with **V** = **ZG**_2_ ***Z***′*σ*_*u*_^2^ + **WW**′*σ*_*pe*_^2^ + **I**_*M*_ *σ*_*e*_^2^. In this scenario, the significant effect of the SNP marker can be tested using a standard Wald statistics computed from the ratio of the estimate of *β*_*SNP*_ and its standard error. However, the application of this test across the whole genome is computationally prohibitive. Alternatively, the association of a given SNP with SCR can be evaluated in a more computationally efficient way using the following test statistic,

$$ \boldsymbol{z}=\frac{{\mathbf{X}}_{\mathbf{SNP}}^{\mathbf{\prime}}{\mathbf{V}}_{\mathbf{o}}^{-1}\left(\mathbf{y}-\mathbf{X}\widehat{\boldsymbol{\upbeta}}\right)}{\sqrt{{\mathbf{X}}_{\mathbf{SNP}}^{\mathbf{\prime}}{\mathbf{V}}_{\mathbf{o}}^{-1}{\mathbf{X}}_{\mathbf{SNP}}}} $$

which approximates the Wald test, and hence, is asymptotically standard normal. Here, **V**_**o**_ is computed as **V** but from a model where the term *X*_*SNP*_ *β*_*SNP*_ is removed, and $\widehat{\boldsymbol{\upbeta}}$ is obtained from the model **y** = **Xβ** + *X*_*SNP*_ *β*_*SNP*_ + **e**, assuming **e** ∼ *N*(0, **V**_***o***_σ_e_^2^). These analyses were performed using the *R* package *RepeatABEL* [29].

### Gene set analysis

The gene set analysis consists basically in three different steps [24, 30]: (i) the assignment of SNPs to genes, (ii) the assignment of genes to functional categories, and finally (iii) the association analysis between each functional category and the phenotype of interest.

1. The SNPs were assigned to bovine genes based on the UMD3.1 bovine genome sequence assembly [31] using the Bioconductor *R* package *biomaRt* [32, 33]. A given SNP was assigned to a particular gene if it was located within the gene or at most 15 kb either upstream or downstream the gene. An arbitrary threshold of *P*-value ≤ 0.01 was used to define significant SNPs (based on the results of the cGWAS); in this context, significant genes were defined as those genes that contained at least one significant SNP.
2. The databases Gene Ontology (GO) [34], and Medical Subject Headings (MeSH) [35, 36] were used to define functional categories of genes. The idea is that genes assigned to the same functional category can be considered as members of a group of genes that share some particular properties, typically their involvement in the same biological or molecular process.
3. The significant association of a given term with SCR was analyzed using Fisher’s exact test. The *P*-value of observing *g* significant genes in the term was calculated by
  $$ Pvalue=1-{\displaystyle \sum_{i=0}^{g-1}}\frac{\left(\begin{array}{c}\hfill S\hfill \\ {}\hfill i\hfill \end{array}\right)\left(\begin{array}{c}\hfill N-S\hfill \\ {}\hfill k-i\hfill \end{array}\right)}{\left(\begin{array}{c}\hfill N\hfill \\ {}\hfill k\hfill \end{array}\right)} $$
  where *S* is the total number of significant genes associated with SCR, *N* is the total number of genes that were analyzed, and *k* is the total number of genes in the term considered [24, 37]. The GO gene set enrichment analysis was performed using the *R* package *goseq* (using method hypergeometric) [38] while the MeSH enrichment analysis was carried out using the *R* package *meshr* [39, 40]. Additionally, the semantic similarities among GO functional terms were calculated based on the GO hierarchy using the *R* package *GOSemSim* [41].

## Results

### Whole genome association analysis

**Fig. 1:**
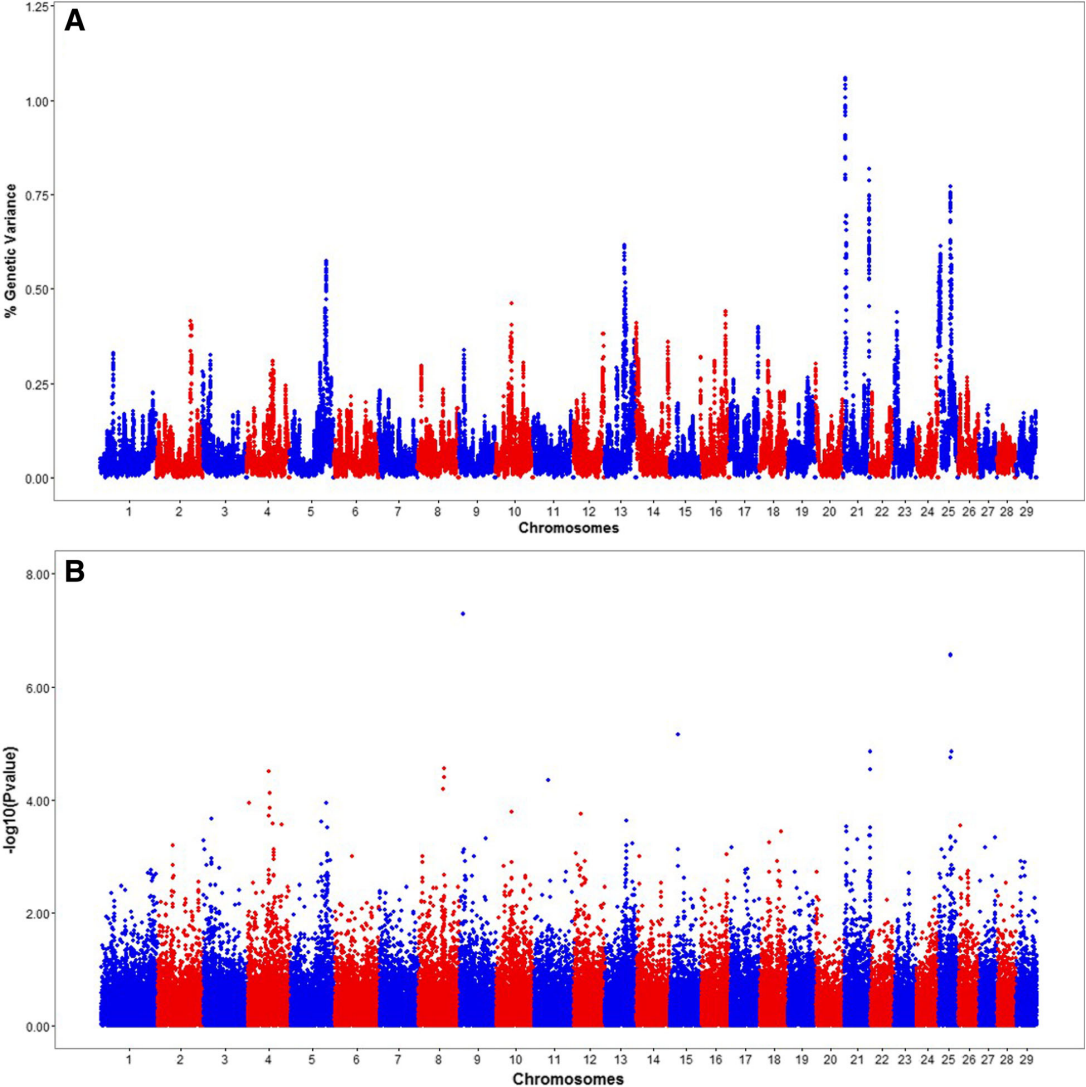
Manhattan plots showing the results of the genome-wide association mapping for Sire Conception Rate: **a** Percentage of genetic variance explained by 1.5 Mb SNP windows across the genome (ssGBLUP method), and **b** − *log*_10_(*Pvalue*) for each of the genetic markers evaluated across the genome (cGWAS method)

Two complementary genome-wide association approaches, ssGBLUP and cGWAS, were performed in order to identify genomic regions and candidate genes associated with Sire Conception Rate (*ĥ* ^2^ = 0.32). These two alternative methods slightly differ in how they identify significant regions or genes associated with the phenotype of interest. On the one hand, ssGLUP allows to identify genomic regions that explain a given amount of genetic variance. On the other hand, using cGWAS, it is possible to formally evaluate the significance of the association (using a statistical test) between each genetic marker and the phenotype of interest. In our study, these two methods yielded very similar results; in fact, the spearman’s rank correlation coefficient between the SNP effects calculated with ssGLUP and cGWAS was equal to 0.943. In addition, the corresponding Manhattan plots showed similar profiles with common significant regions in BTA21 and also BTA25 (Fig. 1). Note that, as expected, ssGBLUP yields less noisy results with well-defined peaks across the entire genome.

**Fig. 2:**
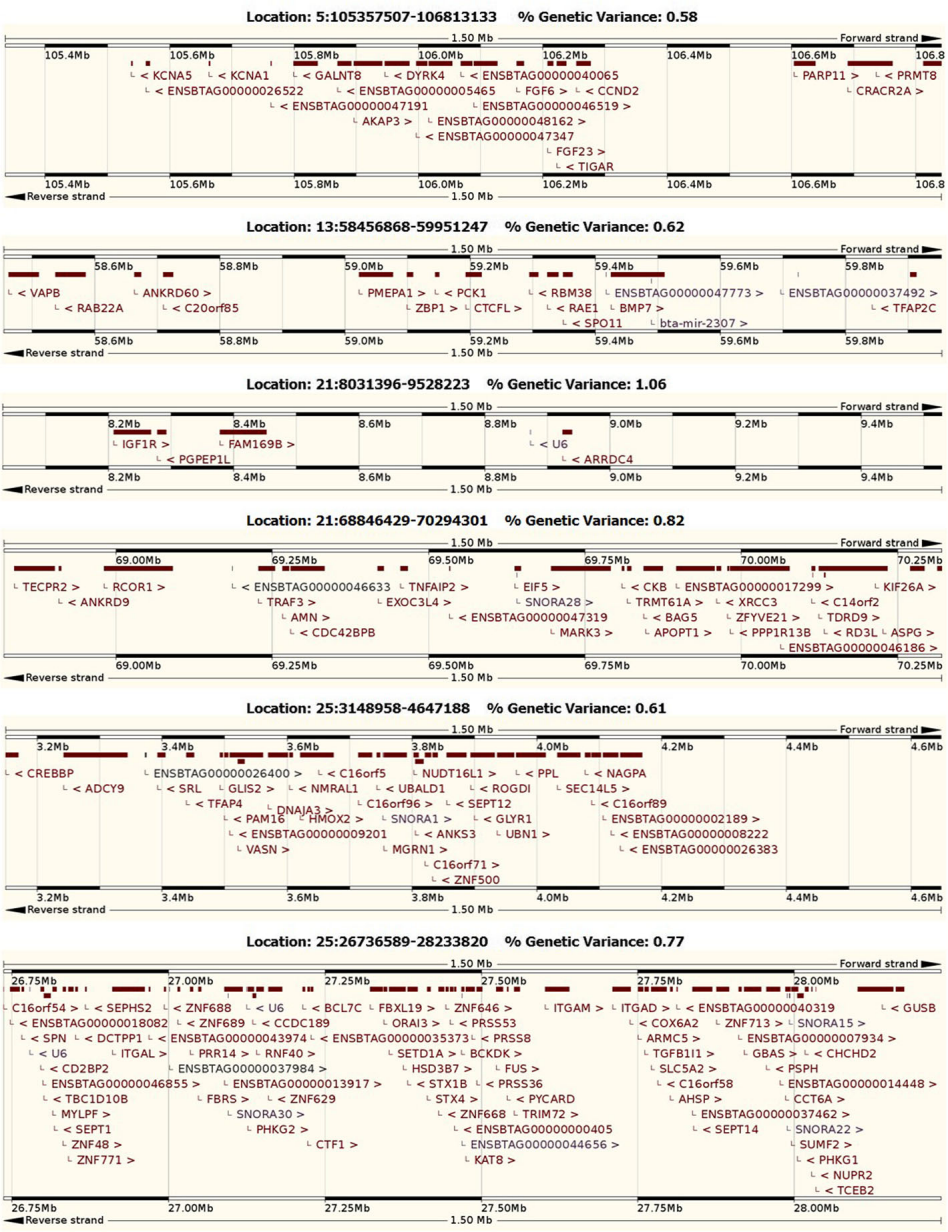
Genomic regions (1.5 Mb) that explain more than 0.50 % of the genetic variance for Sire Conception Rate: genomic location, percentage of variance explained, and list of genes. Adapted from www.ensembl.org using bovine assembly UMD 3.1

Figure 1a displays the results obtained with ssGLUP method in terms of the proportion of genetic variance explained by 1.5 Mb SNP windows across the entire bovine genome. A total of six different genomic regions, distributed on chromosomes BTA5, BTA13, BTA21 and BTA25, explained more than 0.50 % of the genetic variance for sire conception rate. Figure 2 shows the genomic location, the percentage of genetic explained, and the list of genes located in each of these SNP windows. The region that explained the highest percentage of genetic variance (1.06 %) was located on chromosome 21 (21:8031396–9528223). Interestingly, this region harbors *IGF1R*, an insulin-like growth factor receptor that plays critical roles in different reproductive events, including testis development and spermatogenesis. Another SNP-window on BTA21 (21:68,846,429-70,294,301) explained also a substantial amount of genetic variance (0.82 %); this regions harbors two genes, *TDRD9* and *CKB*, which are implicated in sperm development and sperm quality, respectively. Moreover, two different regions on BTA25 (25:3148958–4647188, and 25:26736589–28233820) explained together almost 1.50 % of the genetic variance. Notably, these regions harbor several putative candidate genes for bull fertility, including *MGRN1* and *SEPT12*, which are directly involved in spermatogenesis, and *CCT6A* that is implicated in the fertilization process. Finally, two genomic regions on BTA5 and BTA13 were also identified; each of these windows explains roughly 0.60 % of the genetic variance. The region located on BTA5 (5:105357507–106813133) harbors two genes, *PARP11* and *AKAP3*, that are involved in sperm maturation and motility. In addition, at least two putative genes related to male infertility, *CTCFL* and *SPO11*, are located in the middle of the region detected on BTA13 (13:58456868–59951247).

Figure 1b displays the results obtained with cGWAS in terms of − *log*_10_(*Pvalue*) for each of the SNP markers evaluated across the genome. In addition, Table 1 describes in detail the six most significant SNP markers detected in this analysis (*P*-value ≤ 1.5 × 10^− 5^; *q*-value ≤ 0.15). The most significant SNP (*BTB-01438088*, *P*-value = 5.1 × 10^− 8^) is located in BTA9 in an intron of the gene *RIMS1*. This gene regulates synaptic vesicle exocytosis and is also involved in the regulation of voltage-gated calcium channels. Unsurprisingly, the *RIMS1* allele negatively associated with conception rate is in low frequency in the population (*f*_B_ = 0.038). Two SNP markers located in chromosome 25, *BTA-59768-no-rs* and *ARS-BFGL-NGS-112660*, showed remarkable associations with sire conception rate (*P*-value = 2.8 × 10^− 7^). Note that this genomic region (BTA25 26–28 Mb) was also detected using ssGLUP method. The two significant markers were highly correlated (high linkage disequilibrium), and therefore, it is very likely that they represent the same genetic signal. The marker *BTA-59768-no-rs* is located in an intron of the gene *KAT8*. This gene encodes a histone acetylase implicated in chromatin modification and gene expression regulation. Finally, like ssGBLUP, the single marker regression also detected the region in BTA21 at 68–71 Mb as significantly associated with sire fertility (*P*-value = 1.4 × 10^− 5^). The significant SNP marker *ARS-BFGL-NGS-106232* is located within the gene *BRF1*, which encodes one the subunits of the RNA polymerase III transcription factor complex, and hence, it is directly involved in transcription initiation.

**Table 1:** Most significant genetic markers associated with Sire Conception Rate (SCR)

Marker	Chr	Position	Frequency	β ± se	*P*-value	q-value	Nearest gene
BTB-01438088	9	11867269	0.038	−0.65 ± 0.12	5.1 × 10^− 8^	0.001	RIMS1 (within)
BTB-01138539	15	26472899	0.815	0.26 ± 0.06	7.0 × 10^− 6^	0.102	CADM1 (22 kb)
ARS-BFGL-NGS-106232	21	71210609	0.670	0.20 ± 0.05	1.4 × 10^− 5^	0.136	BRF1 (within)
BTA-59768-no-rs	25	27477941	0.266	−0.29 ± 0.06	2.7 × 10^− 7^	0.005	KAT8 (within)
ARS-BFGL-NGS-112660	25	27672891	0.266	−0.29 ± 0.06	2.8 × 10^− 7^	0.005	ITGAM (34 kb)
Hapmap8541-BTA-59825	25	28711626	0.150	−0.30 ± 0.07	1.4 × 10^− 5^	0.136	TYW1 (within)

### Gene set analysis

The whole-genome association analysis was complemented with a gene set enrichment analysis in order to detect potential functional categories and molecular mechanisms associated with sire fertility. Of the 58,029 SNP markers evaluated in the analysis, 27,066 were located within or surrounding annotated genes; this set of SNPs pointed a total of 17,259 annotated genes. A subset of 349 of these 17,259 genes had at least one SNP with *P*-value ≤ 0.01, and hence, were defined as significantly associated with bull fertility.

**Fig. 3:**
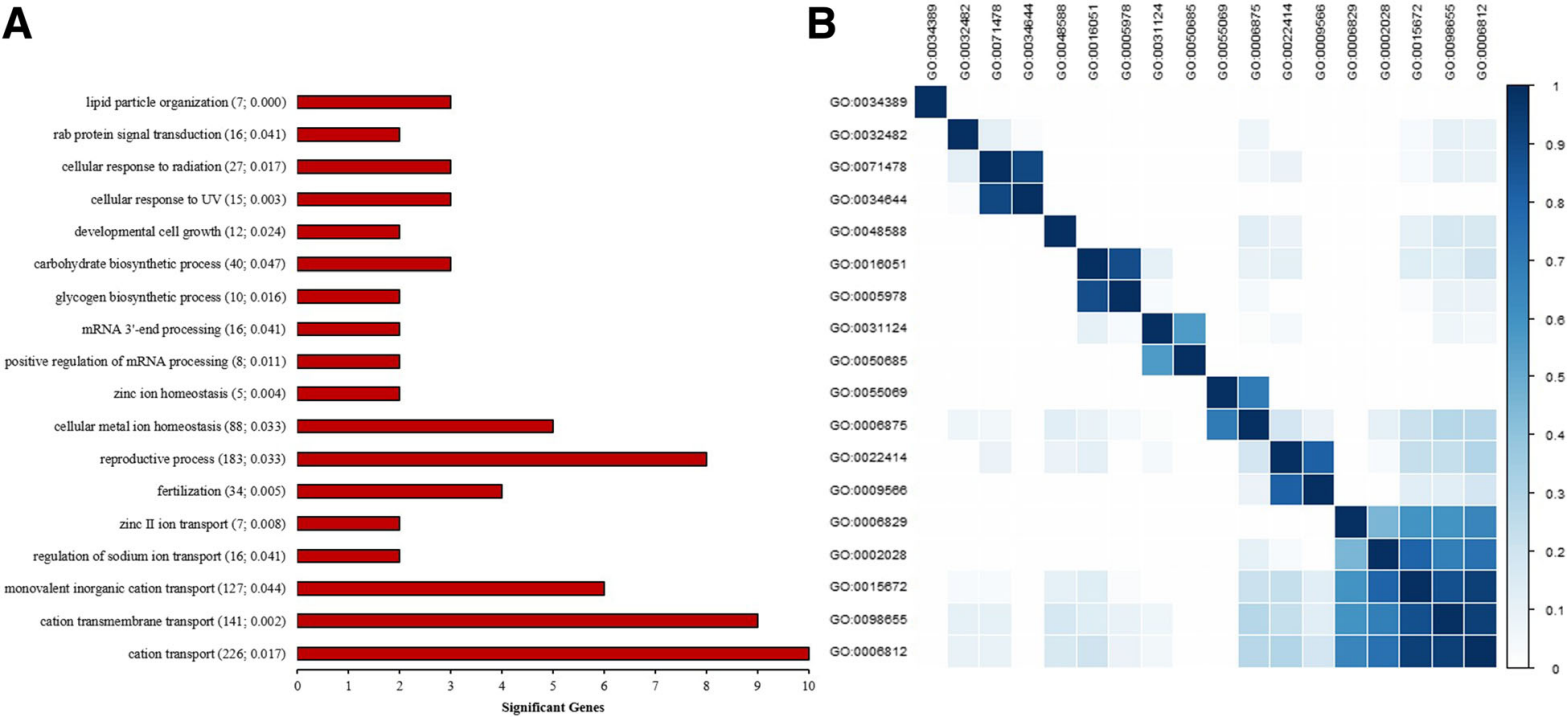
Gene Ontology Biological Process terms significantly enriched with genes associated with Sire Conception Rate: **a** Name, total number of genes, *P*-value, and total number of significant genes per functional term, and **b** Semantic similarity among functional terms

Figure 3 displays a set of GO Biological Process terms that were significantly enriched with genes associated with SCR. Noticeably, some of these terms are closely associated with male fertility, such as *reproduction process* (GO:0022414) and *fertilization* (GO:0009566). These two categories, highly related in the GO hierarchy, had four significant genes in common, namely *BSP3*, *BSP5*, *SLC22A16*, and *ZP2*, all of them directly involved in the process of spermatogenesis and subsequent ovum fecundation. Furthermore, many significant GO terms were associated with ion transport and homeostasis, including *cation transport* (GO:0006812), *zinc II ion transport* (GO:0006829), *regulation of sodium ion transport* (GO:0002028), *zinc ion homeostasis* (GO:0055069), and *cellular metal ion homeostasis* (GO:0006875). Moreover, terms related to developmental biology (e.g. GO:0048588), small GTPase mediated signal transduction (e.g. GO:0032482), and mRNA processing (e.g. GO:0050685) were also enriched with significant genes.

Several GO terms classified into the Molecular Function domain showed an overrepresentation of genes associated with sire fertility (Additional file 2). Especially, functional terms related to channel regulation [e.g., *calcium channel regulator activity* (GO:0005246, *P*-value = 0.020) and *sodium channel regulator activity* (GO:0017080, *P*-value = 0.010)], and transmembrane transporter activity [e.g., *inorganic cation transmembrane transporter activity* (GO:0022890, *P*-value = 0.009) and *ion transmembrane transporter activity* (GO:0015075, *P*-value = 0.015)] showed an overrepresentation of significant genes. Of particular interest, two closely related terms, *SNARE binding* (GO:0000149, *P*-value = 0.007) and *SNAP receptor activity* (GO:0005484, *P*-value = 0.003), which involve a group of membrane-associated proteins that participate in different reproductive events including spermatogenesis and acrosome reaction, were significantly enriched with at least three genes, *STX1A*, *STX1B* and *STX8*, associated with sire conception rate.

Table 2 shows a panel of MeSH terms that were enriched with genes associated with SCR. Many of these terms are closely related to male fertility, such as *spermatozoa* (D013094), *sperm capacitation* (D013075), and *sperm motility* (D013081). Five genes associated with SCR, namely *AKAP3*, *BSP3*, *BSP5*, *NTRK2* and *ZP2*, were part of these terms. Additionally, two other terms related to fertility, *follicle stimulating hormone* (D005640) and *pregnancy rate* (D018873), were also enriched with significant genes, including *AKT1*, *CTTNBP2NL*, *FSHR* and *IGF1R*. Finally, functional categories involving protein kinases (D017868) and GTPases (D020691) were also detected as significant in the MeSH-informed enrichment analysis.

**Table 2:** MeSH terms significantly enriched with genes associated with Sire Conception Rate (SCR)

Mesh term ID	MeSH term name	No. genes	No. significant genes	*P*-value
D005640	Follicle stimulating hormone	34	4	6.4 × 10^− 3^
D013075	Sperm capacitation	9	2	1.6 × 10^− 2^
D013081	Sperm motility	13	4	1.4 × 10^− 4^
D013094	Spermatozoa	71	5	2.0 × 10^− 2^
D017868	Cyclic AMP-dependent protein kinases	75	5	2.5 × 10^− 2^
D018698	Glutamic acid	35	4	7.1 × 10^− 3^
D018873	Pregnancy rate	4	2	2.8 × 10^− 3^
D020691	rab GTP-binding proteins	12	3	2.0 × 10^− 3^

## Discussion

There is growing evidence that bull fertility is influenced by genetic factors. The present study was specifically performed to unravel the genomic architecture underlying sire conception rate, an accurate phenotypic measure of dairy sire fertility. Although previous studies have attempted to identify potential genes and pathways related to SCR [17, 24], this study has some unique features, including the analysis of a large dataset including almost 11 k bulls with about 45 k fertility records, the use of alternative methods for gene mapping, and the application of novel gene set tools, such as MeSH enrichment analysis.

Many methods have been proposed to detect and localize genes underlying complex traits. Given that there is no method that is clearly superior than the others, it is recommended to combine multiple approaches in order to obtain more reliable findings [42]. As such, two alternative whole genome scans were implemented in this study, including a regular single marker regression (cGWAS) and a single-step genomic prediction method (ssGLUP). It is worth noting that these two methods yielded very similar results. In particular, both approaches have identified candidate genomic regions in BTA21 and BTA25 that may be underlying the genetic variation in dairy sire fertility.

The significant region in BTA21 located at 68–71 Mb (see Figs. 1 and 2) harbors at least two candidate genes, namely *CKB* and *TDRD9* that might be directly involved in sire fertility. Gene *CKB* encodes the enzyme creatine kinase, and previous studies have reported that elevated levels of creatine kinase in the sperm are associated with severe oligospermia and male infertility [43]. In fact, some researchers have proposed that creatine kinase should be used as an indicator of sperm quality and maturity in humans [44]. Similarly, gene *TDRD9* encodes an helicase which plays an important role during spermatogenesis by silencing potential transposable elements, and hence, protecting the integrity of the male germline [45]. Hence, our findings provide a foundation for future studies that seek to decipher the specific roles of *CKB* and *TDRD9* in bull fertility. No less important, the results of ssGBLUP in BTA21 at 8–9 Mb strongly suggest *IFGF1* as a candidate gene for sire conception rate. This gene belongs to a family of insulin-like growth factors that has important roles in sex determination, testis development, spermatogenesis and steroidogenesis [46]. Interestingly, *IGF1R* has been implicated in regulating Sertoli cell proliferation and maturation, testis size, and sperm capacitation [47, 48]. Therefore, our findings provide more evidence of the association between *IGF1R* and male fertility.

Both ssGBLUP and cGWAS identified the region in BTA25 at 26–28 Mb as significantly associated with SCR. This region harbors at least two genes, namely *KAT8* and *CCT6A*, with potential roles in dairy sire fertility. The gene *KAT8*, a member of the MYST histone acetyltransferase family, is highly expressed during sperm development [49], and it plays essential roles during early embryonic development [50]. In addition, the gene *CCT6A* encodes a molecular chaperone that mediates the sperm-ooctyte interaction during fertilization [51]. Moreover, the significant region detected in BTA25 but at 3–4 Mb also contains candidate genes for bull fertility, such as *SEPT12* and *MGRN1*. Indeed, *SEPT12* is expressed specifically in the testis and encodes a GTP-binding protein that has been implicated in sperm morphogenesis, sperm motility and male infertility [52, 53]. Likewise, the gene *MGRN1* is widely expressed in the male reproductive system, and recent studies have shown that *MGRN1* knockout in mice results in male infertility, with disruption of hormones secretion and impaired sperm motility [54]. It should be noted that this specific region in BTA25 had been already associated with sire fertility [17]. Overall, our findings provide further evidence for the presence of one or more genes that affect bull fertility in these regions of BTA25. Additional functional studies, including resequencing and fine mapping, are needed to decipher the roles that these genomic regions have in male fertility.

Given that whole-genome scans only detect the most significant regions, and these regions explain only a small fraction of the genetic variance, additional approaches are needed in order to dissect the complex genetic architecture of a quantitative trait. In the present study, different pathway-based approaches, using GO and MeSH databases, were used in order to obtain additional insights regarding the genetic determinants and biological mechanisms underlying sire fertility. Interestingly, some biological processes directly related to male fertility, such as fertilization and sperm motility, were among the most significant functional categories. Further analyses revealed that at least six genes associated with SCR, including *AKAP3*, *BSP3*, *BSP5*, *NTRK2*, *SLC22A16*, and *ZP2*, were part of these functional categories. Interestingly, the gene *AKAP3* is expressed in the spermatozoa and is involved in sperm motility, sperm capacitation, and the acrosome reaction [55]. In addition, the genes *BSP3* and *BSP5* are two binder of sperm proteins implicated in sperm capacitation and fertilization [56]. The gene *ZP2* encodes a sperm receptor that mediates gamete recognition during the fertilization [57]. These findings clearly demonstrate that gene set tools can greatly complement genome-wide association studies in order to understand the genetic basis of complex traits.

Of special interest, GO molecular function terms related to SNARE proteins showed an overrepresentation of significant genes. SNARE proteins are implicated in membrane fusion events, including several events that occur during spermatogenesis and also the acrosome reaction [58]. In fact, it was proposed that SNARE proteins are key players involved in controlling the acrosome reaction during fertilization [59]. Therefore, our findings provide further evidence regarding the active role of SNARE proteins in male fertility. On the other hand, several GO terms associated with ion transport and channel regulation also showed a significant enrichment of genes associated with SCR. It is well-documented that ion channels regulate several sperm physiological responses, including maturation, motility, and chemotaxis [60]. Interestingly, most of the significant terms were related to calcium transport and regulation, and several studies have reported that calcium is indeed implicated in the regulation of sperm motility, and it is an essential second messenger for the acrosome reaction [61]. Therefore, our findings provide further evidence of the important association between calcium and sperm physiology. More in general, note that the genetic markers located in genes initially detected in our GO or MeSH-informed enrichment analysis may facilitate the incorporation and implementation of genomic selection in commercial breeding schemes.

## Conclusions

In this study, a comprehensive genomic analysis was performed with the purpose of unravelling the genetic architecture underlying sire conception rate in Holstein dairy cattle. Genomic regions in BTA5, BTA9, BTA13, BTA15, BTA21 and BTA25 were associated with sire fertility. Most of these regions harbor genes with known roles in sperm biology, including sperm maturation, motility and fertilization. Moreover, gene set analysis revealed that many of the significant terms, such as reproductive process, calcium ion channels, and SNARE proteins, are implicated in biological processes related to male fertility. Overall, this integrative study sheds light on the genetic variants and mechanisms underlying this complex phenotype in cattle. In addition, these findings can provide opportunities for improving bull fertility via marker-assisted selection.

## Additional files

Additional file 1: Descriptive statistics for Sire Conception Rate (SCR): (A) Distribution of SCR values per evaluation, and (B) Distribution of the total number of SCR records per bull (number of repeated measurements). (JPG 135 kb)
Additional file 2: Gene Ontology Molecular Function terms significantly enriched with genes associated with Sire Conception Rate. (DOCX 23 kb)

## Abbreviations

GEBV: Genomic estimated breeding value
GO: Gene Ontology
GWAS: Genome-wide association studies
MeSH: Medical Subject Headings
SCR: Sire Conception Rate
SNP: Single nucleotide polymorphism
ssGLUP: Single-step genomic best linear unbiased prediction
