# Supplementary figures and images for: Unravelling the genomic architecture of bull fertility in Holstein cattle
Source: BMC Genet. 2016 Nov 14;17:143. doi: 10.1186/s12863-016-0454-6 (PMC5109745; doi:10.1186/s12863-016-0454-6)

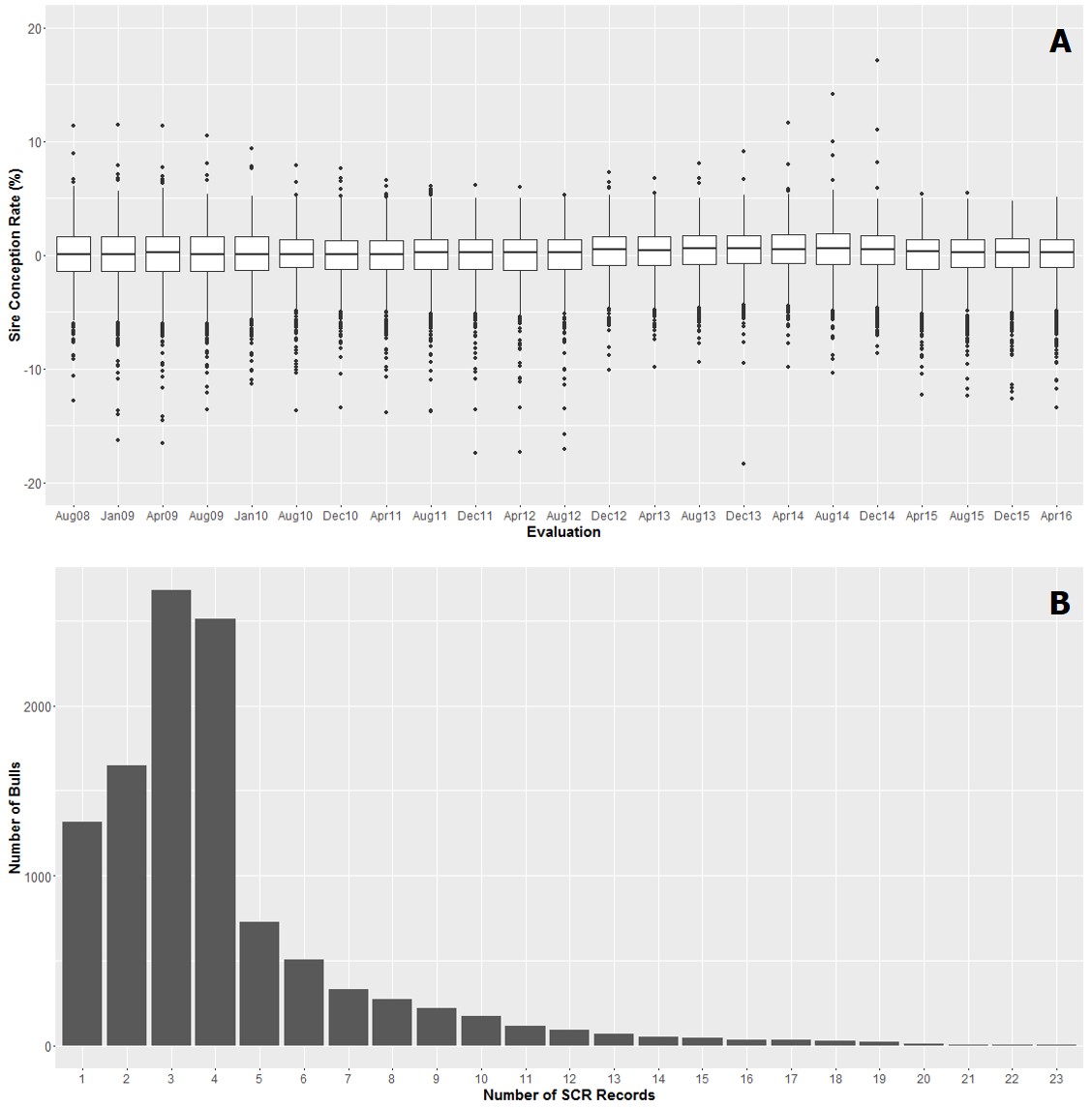

Supplement: Additional file 1: — Descriptive statistics for Sire Conception Rate (SCR): (A) Distribution of SCR values per evaluation, and (B) Distribution of the total number of SCR records per bull (number of repeated measurements). (JPG 135 kb) [file 12863_2016_454_MOESM1_ESM.jpg]
