# Supplementary material for: Unravelling the genomic architecture of bull fertility in Holstein cattle
Source: BMC Genet. 2016 Nov 14;17:143. doi: 10.1186/s12863-016-0454-6 (PMC5109745; doi:10.1186/s12863-016-0454-6)
Supplement: Additional file 2: — Gene Ontology Molecular Function terms significantly enriched with genes associated with Sire Conception Rate. (DOCX 23 kb) [file 12863_2016_454_MOESM2_ESM.docx]

**Additional File 2**. Gene Ontology (GO) Molecular Function terms significantly enriched with genes associated with Sire Conception Rate

| **GO ID** | **MeSH Term Name** | **No. Genes** | **No. Significant Genes** | **P-value** |
| --- | --- | --- | --- | --- |
| GO:0000149 | *SNARE binding* | 20 | 3 | 0.007 |
| GO:0005484 | *SNAP receptor activity* | 15 | 3 | 0.003 |
| GO:0005246 | *calcium channel regulator activity* | 11 | 2 | 0.020 |
| GO:0016247 | *channel regulator activity* | 36 | 5 | 0.001 |
| GO:0017080 | *sodium channel regulator activity* | 8 | 2 | 0.010 |
| GO:0005385 | *zinc ion transmembrane transporter activity* | 7 | 2 | 0.008 |
| GO:0015075 | *ion transmembrane transporter activity* | 188 | 9 | 0.015 |
| GO:0022857 | *transmembrane transporter activity* | 215 | 9 | 0.031 |
| GO:0022890 | *inorganic cation transmembrane transporter activity* | 116 | 7 | 0.009 |
| GO:0019901 | *protein kinase binding* | 77 | 5 | 0.020 |
| GO:0019899 | *enzyme binding* | 283 | 13 | 0.005 |
| GO:0016772 | *transferase activity* | 197 | 10 | 0.007 |
| GO:0016779 | *nucleotidyltransferase activity* | 46 | 5 | 0.002 |
